# A real-world multicenter study on postoperative adjuvant treatment patterns and prognostic implications following neoadjuvant immunochemotherapy for esophageal squamous cell carcinoma

**DOI:** 10.3389/fimmu.2026.1811836

**Published:** 2026-08-19

**Authors:** Xueyuan Zhang, Hongmei Gao, Jianzhong Cao, Min Gao, Yuanji Xu, Wencheng Zhang, Pudong Qian, Xinyi Liu, Zhichao Fu, Wenbin Shen

**Affiliations:** 1 Department of Radiation Oncology, The Fourth Hospital of Hebei Medical University, Shijiazhuang, China; 2 Department of Radiation, Shijiazhuang People’s Hospital, Shijiazhuang, China; 3 Department of Radiation Oncology, Shanxi Province Cancer Hospital, Cancer Hospital Affiliated to Shanxi Medical University, Taiyuan, China; 4 Shandong Cancer Hospital and Institute, Shandong First Medical University, Shandong Academy of Medical Sciences, Jinan, Shandong, China; 5 Department of Radiation Oncology, Clinical Oncology School of Fujian Medical University, Fujian Cancer Hospital, Fuzhou, China; 6 Key Laboratory of Cancer Prevention and Therapy, Tianjin’s Clinical Research Center for Cancer, Tianjin Medical University Cancer Institute and Hospital, National Clinical Research Center for Cancer, Tianjin, China; 7 Department of Radiation Oncology, Nanjing Medical University Affiliated Cancer Hospital & Jiangsu Cancer Hospital & Jiangsu Institute of Cancer Research, Nanjing, China; 8 Department of Radiation Oncology, The 900th Hospital of the Joint Logistic Support Force of the People’s Liberation Army of China, Fuzhou, China

**Keywords:** beneficial population, esophageal squamous cell carcinoma, neoadjuvant immunochemotherapy, postoperative adjuvant therapy, prognosis, real-world study

## Abstract

**Background:**

With the widespread application of neoadjuvant immunochemotherapy (nICT) in locally advanced resectable esophageal squamous cell carcinoma (ESCC), determining the optimal postoperative adjuvant treatment strategy has become a critical clinical issue. This study aimed to evaluate the efficacy of different adjuvant treatment patterns and identify beneficial populations.

**Methods:**

This was a multicenter real-world study involving 612 ESCC patients from six national centers who underwent R0 resection after nICT. Patients were divided into three groups based on postoperative treatment patterns: Clinical Observation (CO group, n=337), Postoperative Adjuvant Chemotherapy (POCT group, n=80), and Postoperative Adjuvant Chemoimmunotherapy (POICT group, n=195). Study endpoints included overall survival (OS), disease-free survival (DFS), and recurrence patterns.

**Results:**

Multivariate analysis of the whole cohort showed that both POCT and POICT reduced the risk of death (HR = 0.51, P = 0.007; HR = 0.49, P<0.001) and disease progression (HR = 0.48, P = 0.003; HR = 0.48, P<0.001) compared with CO. Subgroup analysis indicated that among patients without pathological complete response (non-pCR), both POCT and POICT significantly improved OS and DFS (P<0.05), with no statistical difference between the two groups. In pCR patients, only POICT showed an OS benefit (P = 0.014). Patients with postoperative pathological stage III benefited in both OS and DFS (P<0.05) from POCT and POICT. After propensity score matching (PSM), the 3-year OS (74.7% vs 79.8%) and DFS (63.1% vs 67.2%) rates between the POCT and POICT groups showed no significant difference (P>0.05). Postoperative adjuvant therapy was an independent factor affecting OS in patients with more advanced yT, yN, and yTNM stages, and an independent prognostic factor for DFS in patients with yN2 stage and advanced yTNM stages. Failure pattern analysis revealed no statistically significant differences in the cumulative incidence of locoregional recurrence or distant metastasis between the POCT and POICT groups (χ²=0.279, 2.828; P = 0.597, 0.093).

**Conclusion:**

For ESCC patients receiving nICT, postoperative adjuvant treatment can provide survival benefits, particularly more pronounced in non-pCR patients and those with advanced postoperative pathological stages. Adding immunotherapy to chemotherapy did not significantly further improve survival outcomes in the overall PSM-matched population; however, in pCR patients, POICT showed OS benefit while POCT did not, suggesting potential differential efficacy according to pathological response.

## 1 Introduction

Esophageal squamous cell carcinoma (ESCC) is a prevalent malignant tumor in China, with distinct geographical clustering, particularly around the Taihang Mountain region (1). Most patients are diagnosed at a locally advanced stage, missing the opportunity for direct curative surgery. Currently, neoadjuvant therapy combined with surgery has become the standard treatment mode for locally advanced resectable ESCC (2). In recent years, the application of immune checkpoint inhibitors (ICIs) has brought revolutionary advances to ESCC treatment (3–6). The phase III ESCORT-NEO trial confirmed (7) that neoadjuvant chemotherapy combined with immunotherapy (nICT) significantly increased the pathological complete response (pCR) rate to 28.0%, far exceeding the 4.7% rate with chemotherapy alone. However, against the backdrop of significant pathological response achieved with nICT, the value and strategic choice of postoperative adjuvant treatment have become new clinical challenges. Currently, there is a lack of high-level evidence guiding the need for and the intensity of adjuvant therapy for R0-resected patients after nICT, and the beneficial populations remain unclear. A study based on MPR-ypN pathological classification found (8) significant prognostic differences among patients with different pathological response statuses after nICT, and varying responses to adjuvant therapy. This study suggested that MPR ypN+ patients were more prone to local recurrence, and adjuvant therapy significantly improved recurrence-free survival (RFS) in this subgroup (HR = 0.38). Another study showed (9) that in non-pCR patients, adjuvant therapy significantly improved OS (P = 0.033) and DFS (P = 0.042), highlighting its potential value in certain subgroups.

Current research on postoperative adjuvant therapy for esophageal cancer has primarily focused on traditional chemotherapy or chemoradiotherapy models (10, 11), providing important references for efficacy evaluation under traditional treatment modes. However, in the era of immunotherapy, optimizing postoperative adjuvant strategies urgently requires new evidence, especially regarding whether patients with different postoperative pathological response statuses can benefit from intensified adjuvant treatment, which remains controversial. Furthermore, accurately identifying patients who truly benefit from adjuvant therapy to avoid overtreatment of those at low recurrence risk while providing sufficient treatment intensity for high-risk patients is a core challenge in current clinical decision-making. In this context, this study systematically analyzed the impact of three postoperative patterns—Clinical Observation (CO), Postoperative Adjuvant Chemotherapy (POCT), and Postoperative Adjuvant Chemoimmunotherapy (POICT)—on patient prognosis in ESCC patients after nICT, using national multicenter retrospective data. Propensity score matching analysis was employed to clarify the characteristics of populations benefiting from POCT and POICT, providing high-level evidence for formulating individualized postoperative adjuvant treatment strategies.

## 2 Materials and methods

### 2.1 Study design and patient population

This was a retrospective, multicenter, real-world study. ESCC patients were consecutively enrolled from six medical centers across China. Winning approval from the Fourth Hospital of Hebei Medical University’s ethics committee.

Inclusion Criteria: Pathologically confirmed esophageal squamous cell carcinoma; clinical stage was locally advanced (according to the 8th edition AJCC staging, cT1-4aN0-3M0); ECOG performance status of 0-1; received at least two cycles of standard neoadjuvant immunochemotherapy followed by radical esophagectomy; achieved R0 resection; postoperatively, according to clinical practice, received one of the following three patterns: ① Observation group (CO group) receiving no postoperative adjuvant therapy, ② Adjuvant chemotherapy group (POCT group) receiving at least one cycle of postoperative adjuvant chemotherapy, ③ Adjuvant chemoimmunotherapy group (POICT group) receiving at least one cycle of postoperative adjuvant chemotherapy combined with immunotherapy; complete clinicopathological data, neoadjuvant treatment efficacy evaluation, and follow-up data were available.

Exclusion Criteria: Non-squamous cell carcinoma pathological type; presence of distant metastasis before neoadjuvant therapy; failure to complete planned neoadjuvant therapy or failure to undergo radical surgery (non-R0 resection); perioperative death (within 30 days after surgery or during hospitalization); severely missing clinical data.

### 2.2 Treatment protocols

Neoadjuvant Immunochemotherapy Regimen: Immune checkpoint inhibitors used included: tislelizumab, pembrolizumab, sintilimab, camrelizumab, and toripalimab. Combined chemotherapy regimens were: albumin-paclitaxel/paclitaxel combined with cisplatin/carboplatin. The number of neoadjuvant treatment cycles was 2-4, administered every 3 weeks.

Surgery: All patients underwent radical esophagectomy, primarily minimally invasive McKeown or Ivor Lewis esophagectomy, within 2–8 weeks after completing neoadjuvant therapy.

Postoperative Adjuvant Treatment Regimens: Observation group: routine follow-up only, no antitumor therapy; Adjuvant chemotherapy group: adjuvant chemotherapy, usually similar or identical to the neoadjuvant chemotherapy regimen(albumin-paclitaxel/paclitaxel combined with cisplatin/carboplatin), typically consisting of 4–6 cycles administered every 3 weeks; Adjuvant chemoimmunotherapy group: postoperative adjuvant chemoimmunotherapy, typically consisting of 4–6 cycles of the same immune checkpoint inhibitor combined with chemotherapy as used in the neoadjuvant phase, with no maintenance therapy administered thereafter. The exact number of cycles was individualized based on patient tolerance and postoperative recovery status.

### 2.3 Efficacy and pathological evaluation

Pathological Response Assessment: Postoperative surgical specimens were evaluated blindly by at least two experienced pathologists. Discrepancies were resolved through consensus. Pathological complete response (pCR) was defined as the absence of viable tumor cells in the primary tumor site and all examined lymph nodes. Major pathological response (MPR) was defined as residual viable tumor cells constituting ≤10% of the tumor bed. Tumor regression grade (TRG) was assessed using the Ryan criteria: Grade 0 (complete response): no viable cancer cells; Grade 1 (moderate response): minimal residual disease; Grade 2 (mild response): obvious residual disease; Grade 3 (poor response): minimal or no tumor regression.

Adverse Event Evaluation: Toxicities during neoadjuvant and adjuvant treatment were recorded and graded according to CTCAE version 5.0. Postoperative complications were graded using the Clavien-Dindo classification system.

### 2.4 Follow-up and endpoint definitions

Follow-up: For the first 2 years postoperatively, follow-up occurred every 3–6 months; from years 3 to 5, every 6 months; thereafter, annually. Follow-up included physical examination, laboratory tests (e.g., blood tests, tumor markers), and imaging (chest/abdomen contrast-enhanced CT, endoscopic ultrasound, etc.).

Study Endpoints: The primary endpoint was overall survival (OS), defined as the time from surgery date to death from any cause or last follow-up. The secondary endpoint was disease-free survival (DFS), defined as the time from surgery date to tumor recurrence, metastasis, or death from any cause.

Failure Pattern Definition: The first recurrence or metastasis event was categorized as: Locoregional recurrence (including anastomotic recurrence, mediastinal or abdominal lymph node recurrence); Distant metastasis (metastasis to organs outside the thorax/abdomen and supraclavicular/cervical lymph node metastasis); Mixed recurrence (simultaneous locoregional recurrence and distant metastasis).

### 2.5 Statistical analysis

Categorical variables were expressed as frequencies and percentages, compared using chi-square or Fisher’s exact tests. Normally distributed continuous variables were expressed as mean ± standard deviation, compared using t-tests or ANOVA. Kaplan-Meier method was used to plot survival curves, and Log-rank test was applied for group comparisons. Cox proportional hazards regression model was used to calculate hazard ratios (HR) and 95% confidence intervals (CI) to identify independent prognostic factors for OS and DFS. To reduce bias from imbalanced baseline characteristics, propensity score matching (PSM) analysis (1:N) was performed between the POCT and POICT groups. Subgroup analyses were conducted in the entire cohort and the PSM-matched cohort based on key patient variables, with forest plots drawn to explore potential beneficial populations for different adjuvant treatment patterns. All statistical analyses were performed using R language version 4.5.0 and SPSS version 26.0. A two-sided P<0.05 was considered statistically significant.

## 3 Results

### 3.1 Patient baseline characteristics

**Figure 1:**
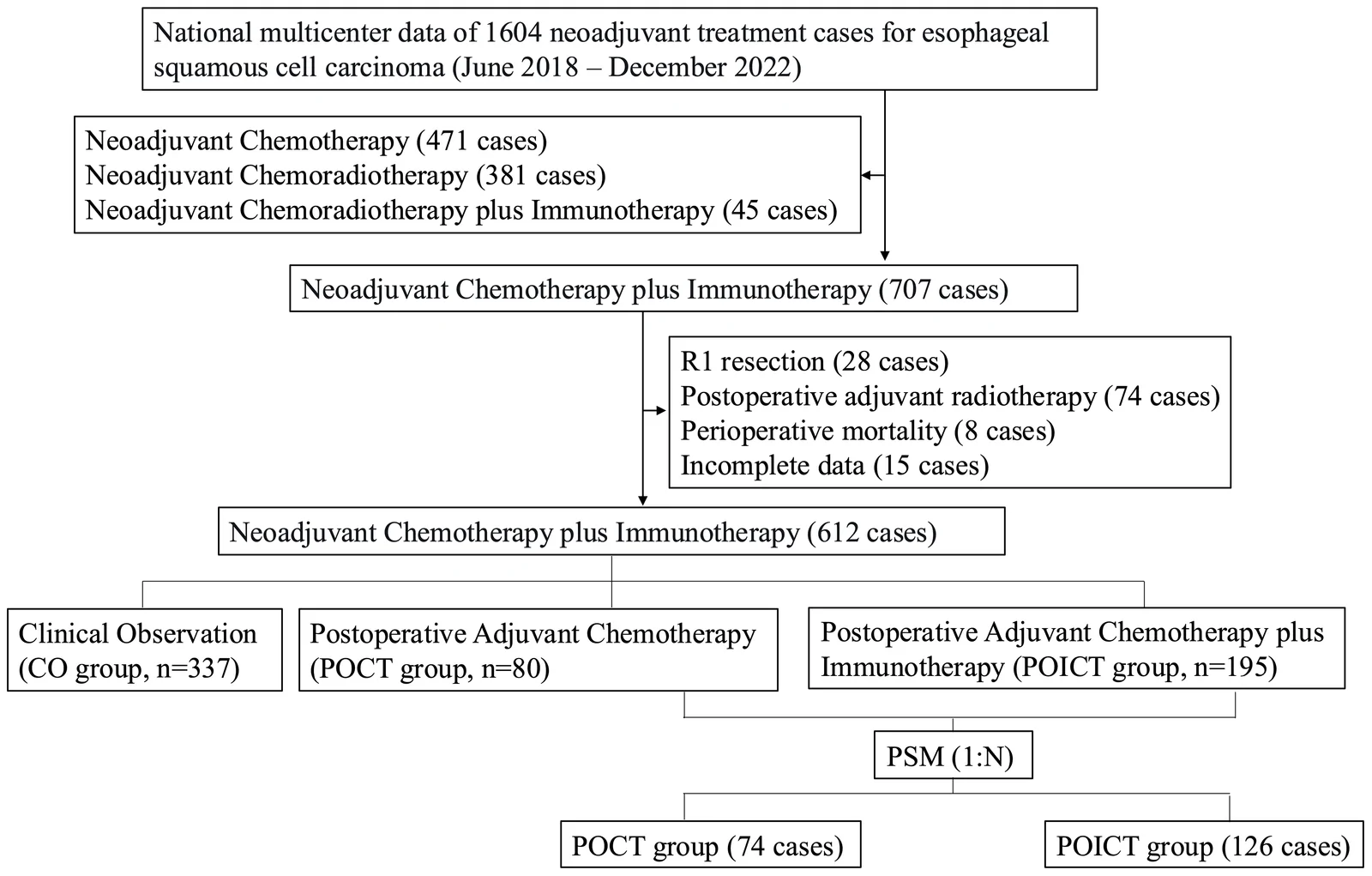
Flow chart of case selection.

A total of 1604 ESCC patients who received neoadjuvant therapy were collected, and 612 patients meeting the inclusion/exclusion criteria were finally included in the analysis (**Figure 1**). The male-to-female ratio was 3.9:1; median age was 64 years (range 43-78); 85.6% had ECOG PS 0. Tumor location was upper thoracic in 12.1%, middle thoracic in 52.3%, and lower thoracic in 35.6%. The numbers of patients receiving 2, 3, and 4 cycles of neoadjuvant chemoimmunotherapy were 390, 173, and 49, respectively. The intervals between neoadjuvant therapy completion and surgery were <4 weeks in 19.3%, 4–6 weeks in 50.3%, and >6 weeks in 30.4% of patients. The incidence of postoperative complications (Clavien-Dindo grade ≥2) was 17.8% in the CO group, 15% in the POCT group, and 11.8% in the POICT group, with no significant difference among the three groups (P = 0.181). Postoperative ypTNM stages were I, II, IIIA, and IIIB in 293, 85, 77 and 157 patients, respectively. Postoperative pCR and MPR rates were 23.0% and 64.5%, respectively. Postoperative adjuvant treatment patterns were no adjuvant therapy in 337, adjuvant chemotherapy in 80, and adjuvant chemoimmunotherapy in 195 patients. The median number of cycles was 4 (range 2–6) in the POCT group and 4 (range 2–6) in the POICT group. In the POICT group, the distribution of specific ICIs was as follows: tislelizumab in 37.4% of patients, pembrolizumab in 22.6%, sintilimab in 17.4%, camrelizumab in 11.8%, and toripalimab in 10.8%. Details are shown in **Table 1**.

**Table 1:** Demographic and clinical characteristics of the entire cohort stratified by postoperative treatment modalities.

Items	Postoperative treatment modalities N(%)			X^2^	P
	No	Chemotherapy	Chemoimmunotherapy		
Gender				2.178	0.337
Male	262 (53.7)	64 (13.1)	162 (33.2)		
Female	75 (60.5)	16 (12.9)	33 (26.2)		
Age				14.994	0.001
≤64	156 (48.1)	44 (13.6)	124 (38.3)		
>64	181 (62.8)	36 (12.5)	71 (24.7)		
ECOG				8.865	0.012
0	279 (53.2)	66 (12.6)	179 (34.2)		
1	58 (65.9)	14 (15.9)	16 (18.2)		
Smoking history				0.552	0.759
No	186 (55.0)	47 (13.9)	105 (31.1)		
Yes	151 (55.1)	33 (12.0)	90 (32.9)		
Drinking history				6.061	0.048
No	208 (54.7)	59 (15.5)	113 (29.7)		
Yes	129 (55.6)	21 (9.1)	82 (35.3)		
Lesion location				2.260	0.688
Upper	42 (56.8)	11 (14.9)	21 (28.4)		
Middle	177 (55.3)	45 (14.1)	98 (30.6)		
Lower	118 (54.1)	24 (11.0)	76 (34.9)		
Neoadjuvant cycles of chemoimmunotherapy				12.352	0.002
2	202 (51.8)	65 (16.7)	123 (31.5)		
3~4	135 (60.8)	15 (6.8)	72 (32.4)		
Operation interval				17.289	0.002
<4 weeks	94 (50.5)	38 (20.4)	54 (21.0)		
4~6 weeks	172 (55.8)	37 (12.0)	99 (32.1)		
>6 weeks	71 (60.2)	5 (4.2)	42 (35.6)		
yT stage				4.211	0.648
0	98 (58.3)	15 (8.9)	55 (32.7)		
1	38 (52.1)	11 (15.1)	24 (32.9)		
2	88 (56.4)	22 (14.1)	46 (29.5)		
3	113 (52.6)	32 (14.9)	70 (32.6)		
yN stage				1.984	0.739
0	212 (56.1)	44 (11.6)	122 (32.3)		
1	82 (53.9)	24 (15.8)	46 (30.3)		
2	43 (52.4)	12 (14.6)	27 (32.9)		
ypTNM stage				4.887	0.558
I	170 (58.0)	32 (10.9)	91 (31.1)		
II	42 (49.4)	12 (14.1)	31 (36.5)		
IIIA	38 (49.4)	14 (18.2)	25 (32.5)		
IIIB	87 (55.4)	22 (14.0)	48 (30.6)		
PCR				2.431	0.297
no	255 (54.1)	67 (14.2)	149 (31.6)		
yes	82 (58.2)	13 (9.2)	46 (32.6)		
MPR				1.810	0.405
no	214 (54.2)	57 (14.4)	124 (31.4)		
yes	123 (56.7)	23 (10.6)	71 (32.7)		
TRG				5.366	0.498
0	82 (58.2)	13 (9.2)	46 (32.6)		
1	87 (55.8)	18 (11.5)	51 (32.7)		
2	74 (51.0)	21 (14.5)	50 (34.5)		
3	94 (55.3)	28 (16.5)	48 (28.2)		
Adjuvant cycles of chemoimmunotherapy					
2∼3	–	13 (37.1)	22 (62.9)	1.260	0.262
4∼6	–	67 (27.9)	173 (72.1)		
Clavien-Dindo					
<2	277 (53.6)	68 (13.2)	172 (33.3)	3.421	0.181
≥2	60 (63.2)	12 (12.6)	23 (24.2)		

### 3.2 Survival analysis and prognostic factors

For the entire cohort, the 1-, 2-, and 3-year OS rates were 90.8%, 79.3%, 71.3%, and DFS rates were 80.0%, 65.9%, 60.6%. As shown in **Table 2**, multivariate analysis revealed that postoperative adjuvant therapy was an independent protective factor for OS and DFS. Compared to the CO group, POCT reduced the risk of death by 49% (HR = 0.51, 95% CI: 0.309-0.829, P = 0.007) and disease progression by 52% (HR = 0.48, 95% CI: 0.289-0.781, P = 0.003). POICT reduced the risk of death by 51% (HR = 0.49, 95% CI: 0.339-0.716, P<0.001) and disease progression by 52% (HR = 0.48, 95% CI: 0.331-0.700, P<0.001). Other factors significantly associated with OS or DFS included: Age >64 years was associated with increased risk for both OS (HR = 1.39, P = 0.040) and DFS (HR = 1.39, P = 0.038); Surgery interval 4–6 weeks vs. <4 weeks was associated with reduced OS risk (HR = 0.78, P = 0.004); More advanced ypTNM stage was associated with worse OS (HR = 2.29, P<0.001) and DFS (HR = 2.57, P<0.001).

**Table 2:** Univariate and multivariate analysis of prognostic factors for the entire cohort.

Items	N	OS univariable			DFS univariable			OS Multivariate			DFS Multivariate		
		HR	95.0% CI	P	HR	95.0% CI	P	HR	95.0% CI	P	HR	95.0% CI	P
Gender													
Male	488		Ref.			Ref.			Ref.			Ref.	
Female	124	0.81	0.547-1.189	0.277	0.72	0.512-1.019	0.064	0.96	0.615-1.490	0.847	1.096	0.704-1.705	0.686
Age													
≤64 years	324		Ref.			Ref.			Ref.			Ref.	
>64 years	288	1.24	0.922-1.675	0.154	1.19	0.919-1.532	0.190	1.39	1.016-1.892	**0.040**	1.39	1.018-1.887	**0.038**
ECOG													
0	524		Ref.			Ref.			Ref.			Ref.	
1	88	1.20	0.804-1.786	0.374	1.37	0.975-1.913	0.070	0.99	0.650-1.521	0.980	1.144	0.748-1.750	0.535
Smoking history													
No	338		Ref.			Ref.			Ref.			Ref.	
Yes	274	1.47	1.092-1.989	**0.011**	1.33	1.025-1.713	**0.032**	1.53	0.995-2.338	0.053	1.38	0.906-2.088	0.135
Drinking history													
No	380		Ref.			Ref.			Ref.			Ref.	
Yes	232	1.18	0.870-1.607	0.284	1.19	0.916-1.545	0.194	1.38	0.900-2.122	0.136	1.49	0.971-2.279	0.068
Lesion location													
Upper	74		Ref.			Ref.			Ref.			Ref.	
Middle	320	0.82	0.514-1.294	0.386	0.81	0.547-1.196	0.288	1.098	0.675-1.784	0.707	1.23	0.758-2.005	0.399
Lower	218	1.01	0.628-1.619	0.973	0.88	0.588-1.324	0.546	0.795	0.570-1.109	0.176	0.92	0.659-1.284	0.623
Neoadjuvant cycles of chemoimmunotherapy													
2	390		Ref.			Ref.			Ref.			Ref.	
3~4	222	1.25	0.915-1.695	0.163	1.12	0.863-1.465	0.385	0.923	0.657-1.295	0.642	0.81	0.577-1.131	0.214
Operation interval													
<4 weeks	186		Ref.			Ref.			Ref.			Ref.	
4~6 weeks	308	0.49	0.330-0.714	**<0.001**	0.71	0.511-0.988	**0.042**	0.78	0.582-0.819	**0.004**	0.59	0.379-0.908	**0.017**
>6 weeks	118	0.71	0.475-1.050	0.086	0.72	0.504-1.036	0.077	0.96	0.498-1.223	0.280	0.89	0.569-1.384	0.598
ypT stage													
0	168		Ref.			Ref.			Ref.			Ref.	
1	73	0.85	0.417-1.723	0.647	1.17	0.675-2.017	0.581	0.57	0.208-1.583	0.283	0.63	0.228-1.711	0.360
2	156	1.38	0.816-2.322	0.231	1.71	1.121-2.606	**0.013**	0.93	0.371-2.343	0.881	0.88	0.354-2.198	0.787
3	215	3.93	2.538-6.077	**<0.001**	3.50	2.411-5.067	**<0.001**	2.51	1.017-6.171	**0.046**	2.43	0.994-5.914	0.052
ypN stage													
0	378		Ref.			Ref.			Ref.			Ref.	
1	152	2.12	1.501-2.996	**<0.001**	2.16	1.599-2.908	**<0.001**	1.28	0.513-3.186	0.597	1.05	0.524-2.106	0.890
2	82	3.54	2.420-5.186	**<0.001**	4.08	2.951-5.640	**<0.001**	1.97	0.651-5.962	0.230	1.69	0.690-4.126	0.252
ypTNM stage													
I-II	378		Ref.			Ref.			Ref.			Ref.	
IIIA-IIIB	234	3.69	2.566-5.320	**<0.001**	3.87	2.684-5.569	**<0.001**	2.29	1.471-3.549	**<0.001**	2.57	1.657-3.980	**<0.001**
PCR													
No	471		Ref.			Ref.			Ref.			Ref.	
Yes	141	0.36	0.218~0.579	**<0.001**	0.34	0.224~0.513	**<0.001**	0.52	0.203~1.340	0.176	0.53	0.207~1.358	0.186
MPR													
No	395		Ref.			Ref.			Ref.			Ref.	
Yes	217	0.44	0.307-0.639	**<0.001**	0.45	0.328-0.607	**<0.001**	0.697	0.379-1.280	0.245	0.78	0.425-1.415	0.407
Postoperative adjuvant therapy													
No	337		Ref.			Ref.			Ref.			Ref.	
chemotherapy	80	0.61	0.378-0.980	**0.041**	0.66	0.440-1.003	0.051	0.51	0.309-0.829	**0.007**	0.48	0.289-0.781	**0.003**
chemoimmunotherapy	195	0.52	0.358-0.741	**<0.001**	0.67	0.502-0.904	**0.009**	0.49	0.339-0.716	**<0.001**	0.48	0.331-0.700	**<0.001**

Significant values (P < 0.05) are highlighted in bold.

### 3.3 Subgroup analysis

**Figure 2:**
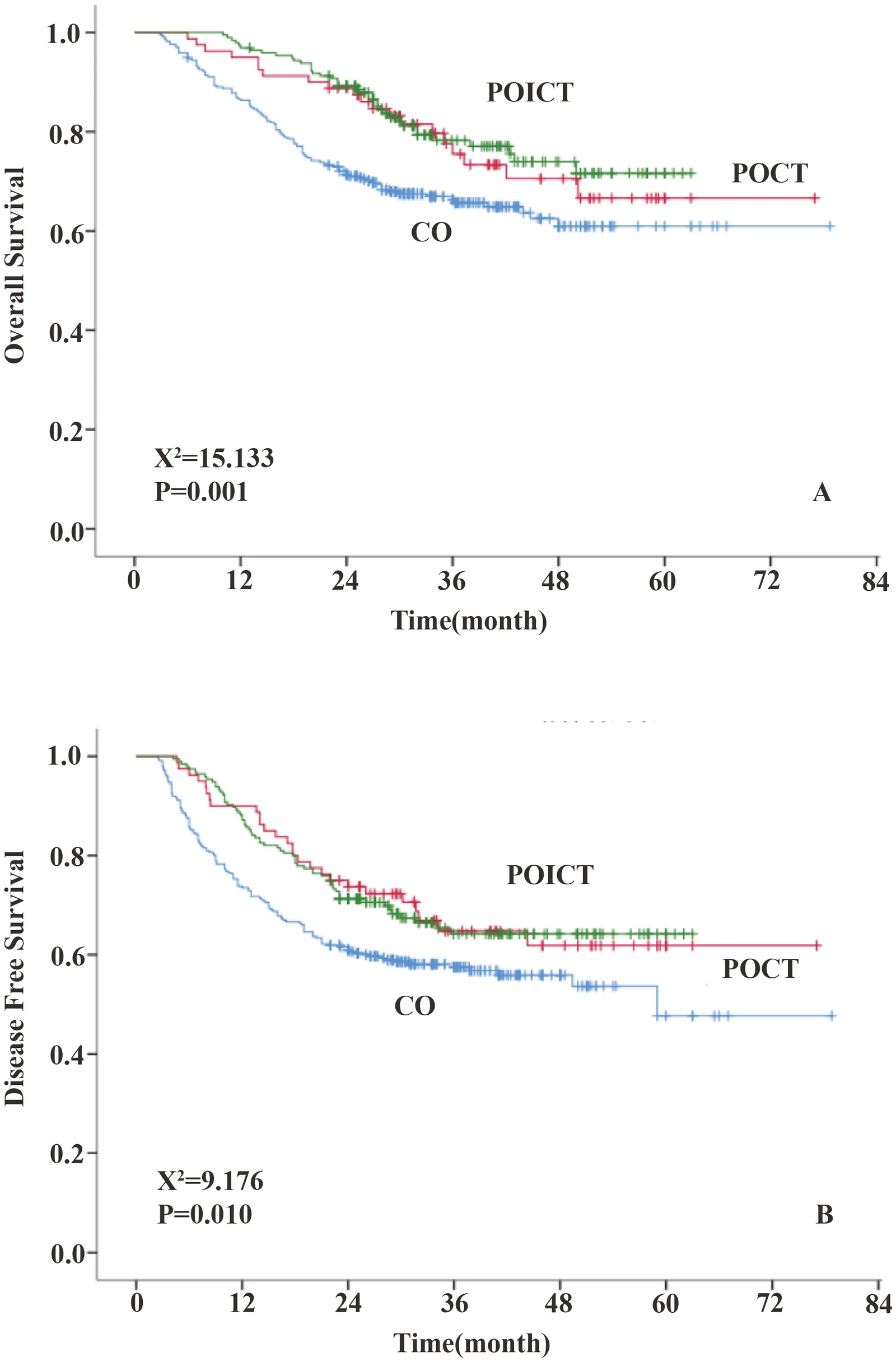
Kaplan-Meier survival curves depicting the impact of three different postoperative adjuvant treatment patterns on patient OS **(A)** and DFS **(B)**.

In exploratory subgroup analyses, patients were divided into three groups based on postoperative adjuvant treatment patterns. Overall comparisons showed statistically significant differences in OS and DFS among the three groups (χ²=15.133, 9.176; P = 0.001, 0.010). Further pairwise comparisons showed that compared to the CO group, both POCT and POICT significantly improved OS (χ²=3.901, 13.228; P<0.05) and DFS (χ²=3.863, 7.132; P = 0.049, 0.008); however, no statistically significant differences were observed in OS or DFS between the POCT and POICT groups. Details are in **Table 3** and **Figure 2**.

**Table 3:** Prognostic analysis of different postoperative adjuvant treatment patterns in the entire cohort.

Group	N	OS (%)			DFS (%)		
		1-year	2-years	3-years	1-year	2-years	3-years
CO	337	86.3	71.4	66.3	73.5	61.0	57.5
POCT	80	95.0	88.8	75.5	90.0	73.7	64.8
POICT	195	96.9	89.2	78.2	87.2	71.3	64.2
X^2^		3.901^a^	13.228^b^	0.186^c^	3.863^a^	7.132^b^	0.010^c^
P		0.048^a^	<0.001^b^	0.668^c^	0.049^a^	0.008^b^	0.922^c^

^a^Comparison between No Adjuvant Therapy and Postoperative Adjuvant Chemotherapy (POCT); ^b^Comparison between No Adjuvant Therapy and Postoperative Adjuvant Chemoimmunotherapy (POICT); ^c^Comparison between POCT and POICT.

**Figure 3:**
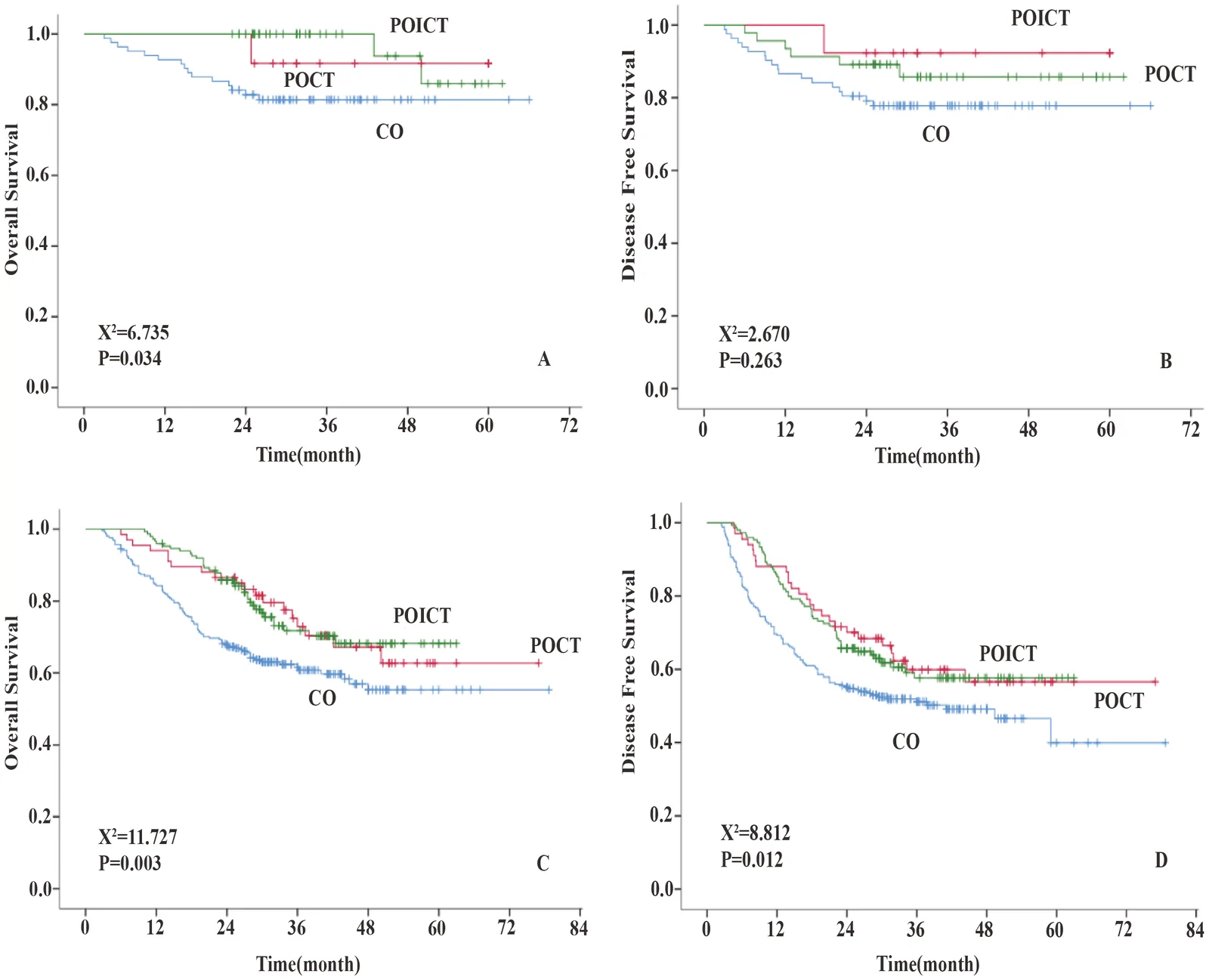
Kaplan-Meier survival curves depicting the impact of three different postoperative adjuvant treatment patterns on OS and DFS in patients with postoperative pathological complete response (pCR) **(A, B)** and non-pCR **(C, D)**.

Analysis of different postoperative adjuvant treatment patterns based on postoperative pCR status: Overall comparison showed statistically significant differences in OS and DFS in the non-pCR subgroup (χ²=11.727, 8.812; P = 0.003, 0.012). In the pCR subgroup, OS difference was significant (χ²=6.735, P = 0.034), but DFS difference was not (χ²=2.670, P = 0.263). Further pairwise comparisons showed that for non-pCR patients, both POCT and POICT groups had significantly better OS (χ²=4.227, 9.468; P = 0.040, 0.002) and DFS (χ²=4.330, 6.357; P = 0.037, 0.012) than the CO group; no significant difference was found between POCT and POICT. For pCR patients, the POICT group had significantly better OS (χ²=6.021, P = 0.014) than the CO group; other pairwise comparisons showed no significant differences. Details are in **Table 4** and **Figure 3**.

**Table 4:** Prognostic impact of different postoperative adjuvant treatment patterns based on pathological response (pCR vs. non-pCR) in the entire cohort.

Group	Non-pCR						pCR					
	OS (%)			DFS (%)			OS (%)			DFS (%)		
	1-year	2-years	3-years	1-year	2-years	3-years	1-year	2-years	3-years	1-year	2-years	3-years
CO	84.3	67.7	61.6	69.3	55.1	51.1	92.7	82.8	81.4	86.6	79.1	77.8
POCT	94.0	86.6	72.9	88.1	70.1	59.9	100.0	100.0	91.7	100.0	92.3	91.7
POICT	96.0	85.8	71.7	85.2	65.8	57.7	100.0	100.0	100.0	93.5	89.1	85.7
X^2^	4.227^a^	9.468^b^	0.001^c^	4.330^a^	6.357^b^	0.081^c^	0.948^a^	6.021^b^	0.206^c^	1.399^a^	1.573^b^	0.309^c^
P	0.040^a^	0.002^b^	0.982^c^	0.037^a^	0.012^b^	0.776^c^	0.330^a^	0.014^b^	0.650^c^	0.237^a^	0.210^b^	0.578^c^

^a^Comparison between No Adjuvant Therapy and POCT; ^b^Comparison between No Adjuvant Therapy and POICT; ^c^Comparison between POCT and POICT.

Analysis of different postoperative adjuvant treatment patterns based on postoperative pathological TNM stage: Results showed statistically significant differences in OS among patients with stage 0, II, and III receiving different postoperative treatments (χ²=6.807, 7.245, 7.601; P = 0.033, 0.027, 0.022), and a statistically significant difference in DFS for stage III patients (χ²=9.579, P = 0.008); other comparisons showed no statistical significance. Details are in **Table 5**.

**Table 5:** Prognostic impact of different postoperative adjuvant treatment patterns based on postoperative pathological TNM stage in the entire cohort.

Group	0 stage			X^2^	P	I stage			X^2^	P	II stage			X^2^	P	III stage			X^2^	P
	1-year	2-years	3-years			1-year	2-years	3-years			1-year	2-years	3-years			1-year	2-years	3-years		
OS				6.807	0.033				0.785	0.675				7.245	0.027				7.601	0.022
CO	92.9	83.2	81.8			94.2	87.2	83.9			77.2	64.6	58.9			80.5	50.6	41.0		
POCT	100.0	100.0	94.7			100.0	94.7	80.7			92.0	84.0	79.1			91.3	82.6	55.3		
POICT	100.0	100.0	100.0			100.0	95.3	86.3			94.5	90.9	76.1			93.9	70.8	56.4		
DFS				2.700	0.259				2.127	0.345				1.414	0.493				9.579	0.008
CO	86.9	79.6	78.3			83.5	74.1	72.8			69.6	59.3	56.8			54.5	31.8	22.6		
POCT	100.0	92.3	92.3			94.7	78.9	78.9			88.6	71.8	56.4			82.6	60.9	46.5		
POICT	93.7	89.6	86.4			95.3	86.0	78.3			85.5	67.3	59.6			75.5	44.9	34.9		

### 3.4 Survival analysis after PSM

**Figure 4:**
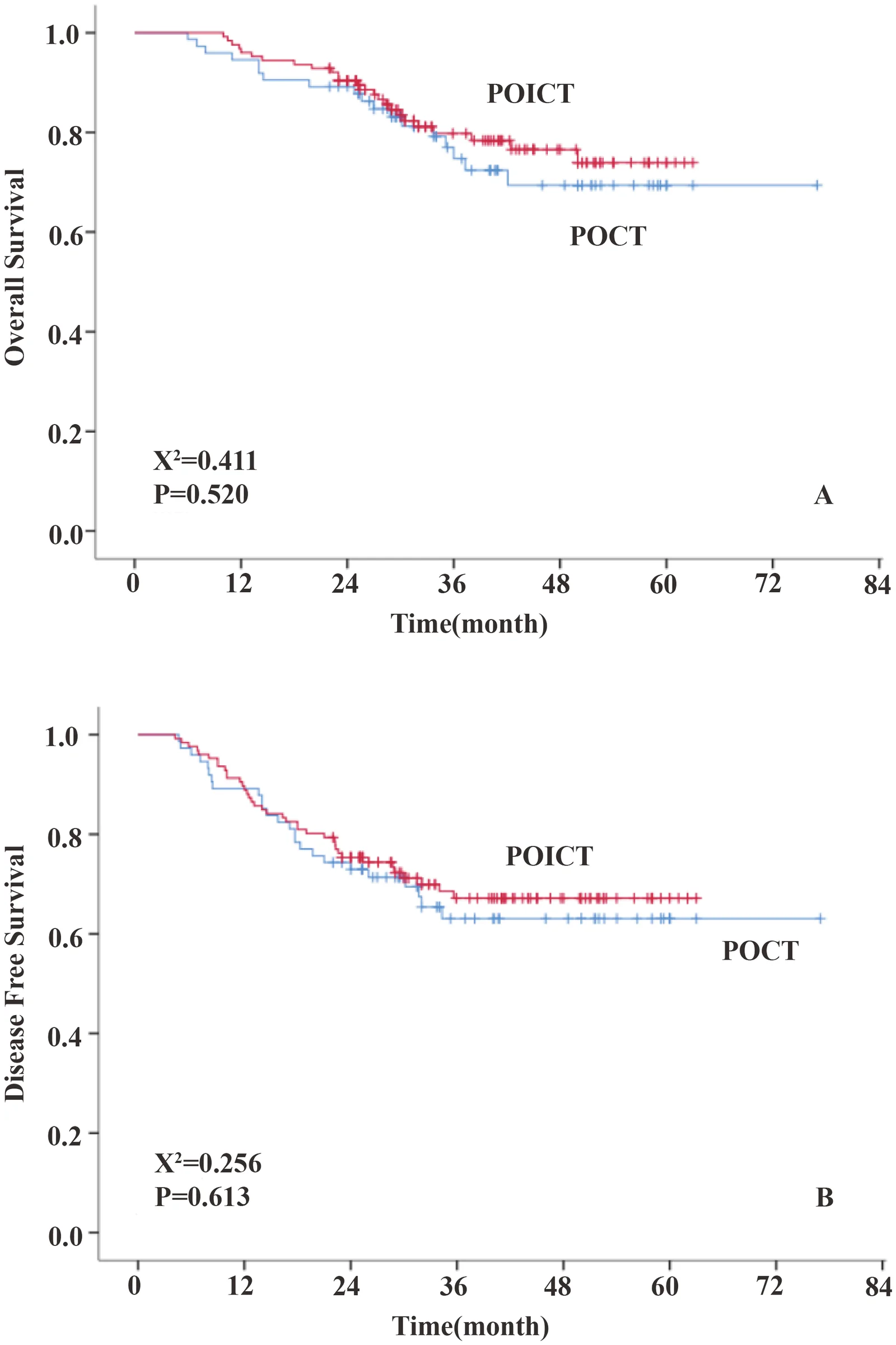
Kaplan-Meier survival curves depicting the impact of two different postoperative adjuvant treatment patterns on OS **(A)** and DFS **(B)** in 200 patients after propensity scorematching (PSM).

**Figure 5:**
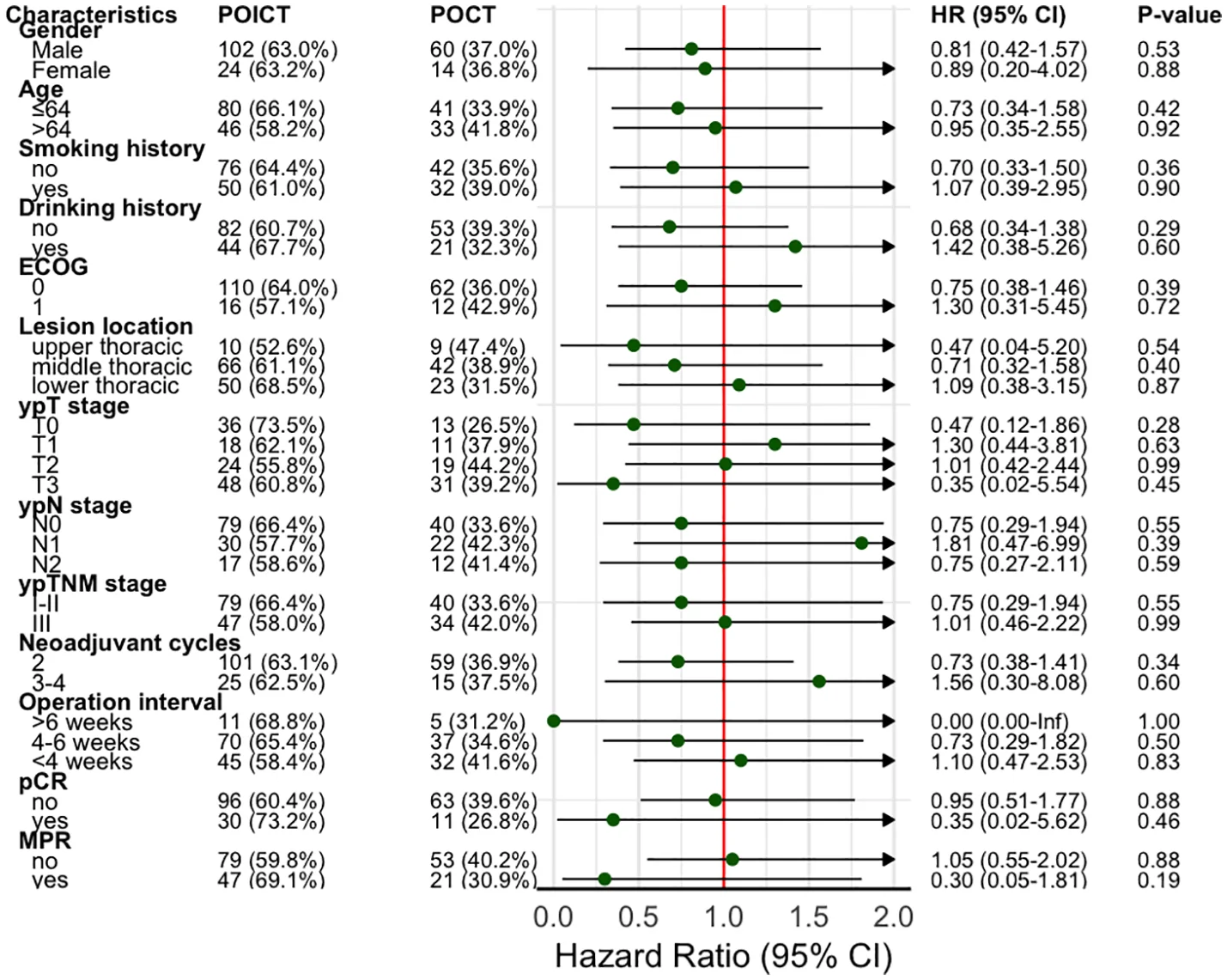
Forest plot of the effect of POCT and POICT on OS across various patient subgroups.

**Figure 6:**
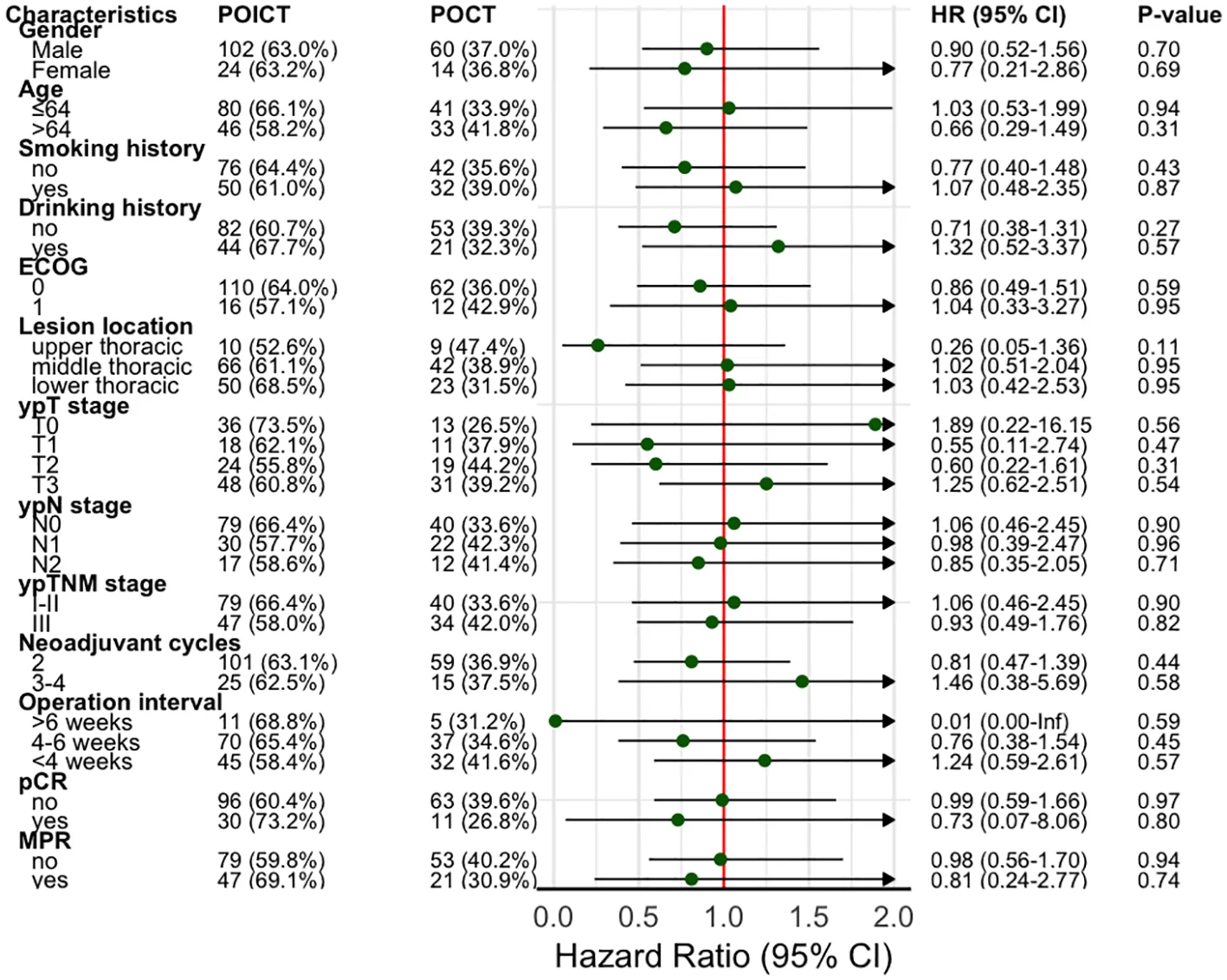
Forest plot of the effect of POCT and POICT on DFS across various patient subgroups.

To assess the impact of POCT versus POICT on patient prognosis, PSM was performed, successfully matching 74 POCT patients with 126 POICT patients (**Table 6**). After PSM, the 1-, 2-, 3-year OS rates for the POCT group were 94.6%, 89.2%, 74.7%, and DFS rates were 89.2%, 72.9%, 63.1%. For the POICT group, the corresponding rates were 96.0%, 90.4%, 79.8% (OS) and 88.9%, 75.4%, 67.2% (DFS). No statistically significant differences were found in OS or DFS rates between the two groups (χ²=0.411, 0.256; P = 0.520, 0.613) (**Figure 4**). Further subgroup analysis of patients receiving POCT or POICT showed no statistically significant impact of either treatment pattern on OS or DFS across various subgroups (**Figures 5**, **6**).

**Table 6:** Demographic and clinical characteristics of patients after propensity score matching (PSM).

Items	Postoperative treatment modalities N(%)		X^2^	P
	Chemotherapy	Chemoimmunotherapy		
Gender			0.001	0.982
Male	60 (37.0)	102 (63.0)		
Female	14 (36.8)	24 (63.2)		
Age			1.276	0.259
≤64	41 (33.9)	80 (66.9)		
>64	33 (41.8)	46 (58.2)		
PS			0.479	0.489
0	62 (36.0)	110 (64.0)		
1	12 (42.9)	16 (57.1)		
Smoking history			0.244	0.621
No	42 (35.6)	76 (64.4)		
Yes	32 (39.0)	50 (61.0)		
Drinking history			0.910	0.340
No	53 (39.3)	82 (60.7)		
Yes	21 (32.3)	44 (67.7)		
Lesion location			1.987	0.370
Upper	9 (47.4)	10 (52.6)		
Middle	42 (38.9)	66 (61.1)		
Lower	23 (31.5)	50 (68.5)		
Neoadjuvant cycles of chemoimmunotherapy			0.005	0.942
2	59 (36.9)	101 (63.1)		
>2	15 (37.5)	25 (62.5)		
Operation interval			1.182	0.554
<4 weeks	32 (41.6)	45 (58.4)		
4~6 weeks	37 (34.6)	70 (65.4)		
>6 weeks	5 (31.3)	11 (68.8)		
yT			3.438	0.329
0	13 (26.5)	36 (73.5)		
1	11 (37.9)	18 (62.1)		
2	19 (44.2)	24 (55.8)		
3	31 (39.2)	48 (60.8)		
yN			1.453	0.484
0	40 (33.6)	79 (66.4)		
1	22 (42.3)	30 (57.7)		
2	12 (41.4)	17 (58.6)		
ypTNM			2.139	0.544
I	28 (32.6)	58 (67.4)		
II	12 (36.4)	21 (63.6)		
IIIA	15 (46.9)	17 (53.1)		
IIIB	19 (38.8)	30 (61.2)		
PCR			2.289	0.130
no	63 (39.6)	96 (60.4)		
yes	11 (26.8)	30 (73.2)		
MPR			1.654	0.198
no	53 (40.2)	79 (59.8)		
yes	21 (30.9)	47 (69.1)		
TRG			3.644	0.303
0	11 (26.8)	30 (73.2)		
1	17 (34.0)	33 (66.0)		
2	20 (39.2)	31 (60.8)		
3	26 (44.8)	32 (55.2)		

Multivariate analysis identified postoperative pathological stage as a key prognostic factor. ypT3 was an independent risk factor for OS (HR = 14.388, P<0.001); ypN2 stage was also significantly associated with both OS and DFS, with HR values of 6.012 (P<0.001) and 8.271 (P = 0.035), respectively. Compared to stage I-II patients, stage IIIA-IIIB patients had significantly increased OS and DFS(HR = 3.025, 3.159; P<0.05) risk. Pathological complete response (pCR) and major pathological response (MPR) were significant protective factors in univariate analysis (HR < 1, P<0.05) but lost significance in multivariate analysis, suggesting their prognostic information might be superseded by more comprehensive postoperative pathological staging. Surgery interval >4 weeks showed a trend towards increased risk (HR>1), but with wide confidence intervals and non-significant P values, possibly due to limited sample size. Details are in **Table 7**.

**Table 7:** Univariate and multivariate analysis of prognostic factors for the 200 patients after PSM.

Items	OS univariable			DFS univariable			OS Multivariate			DFS Multivariate		
	HR	95.0%CI	P	HR	95.0%CI	P	HR	95.0%CI	P	HR	95.0%CI	P
Gender												
Male		Ref.			Ref.			Ref.			Ref.	
Female	0.770	0.343-1.73	0.527	0.641	0.317-1.299	0.217	0.952	0.311~2.918	0.932	0.701	0.320-1.537	0.375
Age												
≤64 years		Ref.			Ref.			Ref.			Ref.	
>64 years	0.829	0.446-1.541	0.554	0.851	0.509-1.422	0.538	0.818	0.358~1.871	0.634	1.233	0.665-2.286	0.507
ECOG												
0		Ref.			Ref.			Ref.			Ref.	
1	1.256	0.582-2.711	0.561	1.585	0.844-2.976	0.152	0.820	0.287~2.338	0.710	1.273	0.659-2.460	0.472
Smoking history												
No		Ref.			Ref.			Ref.			Ref.	
Yes	0.988	0.531-1.836	0.969	1.082	0.655-1.79	0.757	1.145	0.396~3.308	0.803	0.830	0.404-1.706	0.613
Drinking history												
No		Ref.			Ref.			Ref.			Ref.	
Yes	0.867	0.445-1.688	0.674	1.192	0.71-2.001	0.506	1.597	0.532~4.799	0.404	1.102	0.538-2.254	0.791
Lesion location												
Upper		Ref.			Ref.			Ref.			Ref.	
Middle	1.650	0.496-5.481	0.414	0.920	0.408-2.075	0.840	0.583	0.170~2.002	0.391	0.641	0.263-1.563	0.328
Lower	1.717	0.500-5.900	0.390	0.900	0.384-2.108	0.808	0.961	0.510~1.809	0.901	0.593	0.231-1.520	0.276
Neoadjuvant cycles of chemoimmunotherapy												
2		Ref.			Ref.			Ref.			Ref.	
3~4	0.964	0.427-2.177	0.929	0.742	0.377-1.461	0.388	0.964	0.427~2.179	0.931	0.733	0.337-1.596	0.434
Operation interval												
<4 weeks		Ref.			Ref.			Ref.			Ref.	
4~6 weeks	2.077	0.277-15.56	0.477	4.769	0.651-34.93	0.124	3.239	0.403~26.030	0.269	4.036	0.519-31.353	0.182
>6 weeks	3.332	0.448-24.78	0.240	6.039	0.822-44.34	0.077	6.389	0.796~51.254	0.081	4.849	0.624-37.704	0.131
ypT stage												
0		Ref.			Ref.			Ref.			Ref.	
1	2.262	0.378-13.54	0.371	1.629	0.525-5.053	0.398	2.712	0.425~17.283	0.291	0.566	0.109-2.942	0.499
2	4.412	0.937-20.79	**0.060**	3.389	1.326-8.663	**0.011**	5.371	1.074~26.875	**0.041**	0.912	0.195-4.272	0.907
3	11.665	2.784-48.88	**0.001**	4.559	1.916-10.847	**<0.001**	14.388	3.255~63.600	**<0.001**	1.270	0.219-7.351	0.790
ypN stage												
0		Ref.			Ref.			Ref.			Ref.	
1	1.289	0.595-2.793	0.52	1.802	0.983-3.302	0.057	1.336	0.569~3.134	0.506	1.668	0.356-7.818	0.517
2	5.327	2.664-10.652	**<0.001**	5.426	2.993-9.837	**<0.001**	6.012	2.483~14.556	**<0.001**	8.271	1.155-59.238	**0.035**
ypTNM stage												
I-II		Ref.			Ref.			Ref.			Ref.	
IIIA-IIIB	5.797	2.543-13.216	**<0.001**	4.600	2.358-8.977	**<0.001**	3.025	1.279~7.153	**0.012**	3.159	1.327-7.523	**0.009**
PCR												
No		Ref.			Ref.			Ref.			Ref.	
Yes	0.171	0.041-0.709	**0.015**	0.163	0.051-0.521	**0.002**	0.238	0.017~3.426	0.292	0.205	0.030-1.392	0.105
MPR												
No		Ref.			Ref.			Ref.			Ref.	
Yes	0.225	0.088-0.571	**0.002**	0.340	0.177-0.652	**0.001**	1.281	0.296~5.542	0.741	0.967	0.371-2.520	0.946
Postoperative adjuvant therapy												
chemotherapy		Ref.			Ref.			Ref.			Ref.	
chemoimmunotherapy	0.820	0.447-1.504	0.522	0.878	0.530-1.454	0.612	1.034	0.463~2.309	0.934	1.091	0.631-1.886	0.756

Significant values (P < 0.05) are highlighted in bold.

### 3.5 Failure pattern analysis

In the entire cohort, failure occurred in the CO group at 2.5-59.0 months (median 10 months); in the POCT and POICT groups at 3.7-44.3 months (median 14 months). In the CO group, 84 patients (25.0%) experienced treatment failure: isolated locoregional recurrence in 39 (11.6%), distant metastasis in 34 (10.1%), and combined failure in 11 (3.3%). Among the 39 isolated recurrence patients, mediastinal lymph node recurrence was predominant (33 cases), the remaining 6 were anastomotic recurrences. Among the 34 distant metastasis patients: supraclavicular lymph node metastasis (7), lung (8), liver (4), bone (2), brain (1), adrenal (2), abdominal lymph node (3), multi-organ (7). Among the 11 combined failure patients: 2 had anastomotic plus extranodal metastasis, 2 had mediastinal lymph node plus extranodal metastasis, 6 had organ metastasis plus mediastinal lymph node recurrence, and 1 had pleural plus mediastinal lymph node recurrence. In the combined POCT/POICT groups, 74 patients (26.8%) experienced treatment failure: isolated locoregional recurrence in 25 (9.1%), distant organ metastasis in 31 (11.2%), and combined failure in 18 (6.5%). Among the 25 isolated locoregional recurrences: anastomotic (5), mediastinal lymph node (20). Among the 31 distant metastases: neck/supraclavicular lymph node (18), liver (3), abdominal lymph node (2), axillary lymph node (1), and ≥2 organ metastases (7). Among the 18 combined failures: 4 anastomotic recurrence plus organ metastasis, 10 mediastinal lymph node recurrence plus organ metastasis, 4 mediastinal lymph node plus extranodal metastasis.

After PSM: Failure occurred in the POCT group at 7–59 months (median 29 months) and in the POICT group at 4.9–77 months (median 30 months). In the 74 POCT patients: isolated locoregional recurrence in 7 (9.5%), distant metastasis in 2 (2.7%), combined failure in 5 (6.8%). In the 126 POICT patients: isolated locoregional recurrence in 9 (7.1%), distant metastasis in 15 (11.9%), combined failure in 8 (6.3%). The cumulative locoregional failure rates were 16.3% (12/74) for POCT and 13.5% (17/126) for POICT, with no significant intergroup difference (χ²=0.279, P = 0.597). The cumulative distant failure rates were 9.6% (7/74) for POCT and 18.3% (23/126) for POICT, with no significant difference (χ²=2.828, P = 0.093).

## 4 Discussion

This study systematically analyzed the efficacy and beneficial populations of different adjuvant treatment patterns following neoadjuvant immunochemotherapy for ESCC using national multicenter real-world data. The results indicate that postoperative adjuvant therapy, particularly combined immunotherapy, can provide survival benefits for specific populations, offering important evidence for individualized clinical decision-making. This study confirms that postoperative adjuvant therapy remains valuable in the nICT context. Multivariate analysis showed that compared to observation alone, both POCT and POICT significantly improved OS and DFS, reducing the risk of death by approximately 50%. This direction is consistent with the preliminary results of the phase III SKYSCRAPER-07 trial (presented at ESMO 2025) (12), which first demonstrated that consolidation therapy with the PD-L1 inhibitor atezolizumab after definitive chemoradiotherapy significantly improved progression-free and overall survival in patients with unresectable ESCC. Our study extends the applicability of this “immune consolidation” strategy from the unresectable disease setting to the post-neoadjuvant therapy phase in operable patients, further expanding its clinical application.

Our findings are also consistent with several other studies. One study showed that postoperative adjuvant therapy after nICT significantly improved 1- and 2-year PFS in high-risk patients (1-year: 53.3% vs. 26.7%; 2-year: 35.6% vs. 6.7%, P = 0.009) (13). A meta-analysis of 10 studies involving 6462 patients (14) showed that compared to patients receiving neoadjuvant therapy and esophagectomy alone, the adjuvant therapy group had a significantly 48% lower 1-year mortality (RR = 0.52, 95% CI: 0.41-0.65, P<0.001); this mortality reduction persisted up to 5 years of follow-up (RR = 0.91, 95% CI: 0.86-0.96, P<0.001). Notably, this study found that among pCR patients, only POICT significantly improved OS, while POCT showed no significant difference compared to observation. This may be related to the lower recurrence risk in pCR patients, potentially requiring stronger immune activation for further prognostic improvement.

An important finding of this study is the differential response to adjuvant therapy among populations with different pathological responses. In non-pCR patients, both POCT and POICT showed significant survival benefits, with comparable efficacy. These findings should be considered hypothesis-generating and require validation in prospective studies. Residual confounding cannot be excluded due to the non-randomized nature of subgroup assignment.

This finding aligns with previous research. Liu et al. (9) analyzed 662 esophageal cancer patients after neoadjuvant therapy in a real-world study. Subgroup analysis showed that non-pCR patients benefited from postoperative adjuvant therapy in terms of DFS (p=0.042) and OS (p=0.033), noting that adjuvant chemoimmunotherapy provided better survival benefits than adjuvant radiotherapy or chemotherapy alone. Another study by Feng et al. (15) analyzed 215 ESCC patients after nICT, of whom 78 (36.3%) received postoperative adjuvant therapy after R0 resection. Results showed that adjuvant therapy was significantly associated with both DFS (HR: 0.297; P<0.001) and OS (HR: 0.321; P = 0.001) in ypT+N+ patients. Wu et al. (16) studied 112 ESCC patients who received postoperative adjuvant immunotherapy after nICT, showing that the combination of nICT and postoperative adjuvant immunotherapy demonstrated short-term survival benefits with acceptable safety, suggesting it could be an effective strategy for LA-ESCC patients. These studies collectively indicate the critical importance of postoperative adjuvant therapy for patients who do not achieve pCR. Our results are consistent with the implications of the SCIENCE study (17), which showed that neoadjuvant chemoradiotherapy plus sintilimab achieved a pCR rate as high as 47.3%, significantly higher than the 13% with neoadjuvant chemotherapy plus immunotherapy. This suggests that patients who received nICT but did not achieve pCR may still retain some sensitivity to subsequent chemotherapy and immunotherapy within their tumor microenvironment. Given the imbalanced distribution of the various ICIs used in this study, subgroup analyses by ICI type were not performed. Future studies with larger sample sizes are warranted to explore the differences in efficacy among different ICIs.

After balancing baseline characteristics via PSM, this study found no significant difference in survival outcomes between POCT and POICT. This result may suggest that continuing immune checkpoint inhibitors after nICT might not be necessary for all patients. Particularly considering pharmacoeconomics and potential adverse effects, adjuvant chemotherapy alone may be a reasonable alternative for certain patient groups. This finding differs from the traditional concept of “continuing immune checkpoint inhibitor maintenance therapy” and may be related to the durable anti-tumor immune memory potentially induced by nICT. The CROSS and NEOCRTEC5010 trials of neoadjuvant chemoradiotherapy for ESCC (18, 19) both achieved high pCR rates, with pCR patients having favorable prognoses, implying that a strong initial treatment response might reduce the need for subsequent intensive therapy. This aligns with the logic of the RICE phase II study (20) strategy of induction immunochemotherapy followed by concurrent chemoradiotherapy for unresectable patients, where strong initial immune activation might induce durable anti-tumor immune memory, reducing absolute dependence on subsequent continuous immune checkpoint inhibition.

Neoadjuvant immunotherapy combined with chemotherapy or chemoradiotherapy may reduce local and distant recurrence rates and improve long-term survival. Related studies suggest that although nICT might have a poorer pathological response, its survival rates are comparable to neoadjuvant chemoradiotherapy, possibly due to its control of lymph node metastasis (21–23), also indicating that NCRT might not effectively control systemic micrometastasis. The failure pattern analysis in this study showed that failure modes were still dominated by locoregional recurrence and/or distant metastasis. Results also showed that the median recurrence time was shorter in the CO group than in the POCT/POICT groups. After PSM, the median recurrence time for patients receiving adjuvant therapy was over 2 years. There was no significant difference in the cumulative incidence of distant metastasis between POCT and POICT groups (9.6% vs 18.3%, P = 0.093), although the higher numerical value in the POICT group might indicate higher baseline tumor burden or more aggressive biological behavior in this group. This finding emphasizes the need for more precise biomarkers to identify patients at high risk of distant metastasis for targeted intensification of adjuvant therapy. Wang et al. (24) explored recurrence patterns in locally advanced ESCC patients undergoing surgery after nICT. Among 422 patients, 89 (21.1%) experienced recurrence: local recurrence in 37 (8.8%), distant metastasis in 30 (7.1%), combined recurrence in 22 (5.2%). That study also showed that postoperative adjuvant therapy significantly improved local and distant recurrence-free survival in ypN+ patients. Recent research has begun focusing on the relationship between tumor microenvironment characteristics and treatment response. Previous studies (25, 26) found that tertiary lymphoid structure (TLS) density correlates with ESCC patient prognosis, with high TLS density indicating poorer prognosis. Such biomarkers may help identify populations likely to benefit from intensified adjuvant therapy.

As a retrospective real-world study, this research has inherent limitations, including treatment selection bias and lack of standardized treatment protocols. Furthermore, heterogeneity in immunotherapeutic agents and variations in treatment cycles may affect the results. Moreover, systematic adverse event data were lacking because documentation of toxicities (especially low-grade events and reasons for treatment discontinuation) varied across centers, precluding a comprehensive safety assessment. Future prospective randomized controlled trials are needed to further validate our conclusions, especially studies based on biomarker-driven precise classification.

With the deepening application of immunotherapy in esophageal cancer, postoperative adjuvant strategies are gradually shifting from a “one-size-fits-all” approach towards “individualization”. By integrating large-sample, multicenter real-world data, this study confirms the value of adjuvant therapy after nICT for ESCC and reveals differential benefits among populations with different pathological responses. Results show that postoperative adjuvant therapy can provide survival benefits for ESCC patients after nICT, especially for non-pCR populations. Future work needs to further develop reliable biomarker systems for precise identification of beneficial populations; explore novel combination strategies to enhance efficacy in high-risk patients; and optimize treatment intensity and duration to balance efficacy and safety. The ultimate goal is to achieve precise treatment for ESCC patients and maximize clinical benefit. Postoperative adjuvant therapy provides meaningful survival benefits for ESCC patients after nICT. Subgroup analyses suggest potential differential benefits according to pathological response, but these need further confirmation. Final conclusions should be drawn from large-scale randomized controlled studies.

## Data Availability

The raw data supporting the conclusions of this article will be made available by the authors, without undue reservation.
